# A specific combined long-chain polyunsaturated fatty acid supplementation reverses fatty acid profile alterations in a mouse model of chronic asthma

**DOI:** 10.1186/s12944-018-0947-6

**Published:** 2019-01-18

**Authors:** D. Fussbroich, K. Zimmermann, A. Göpel, O. Eickmeier, J. Trischler, S. Zielen, R. Schubert, C. Beermann

**Affiliations:** 1 Department of Food Technology, University of Applied Science Fulda, Leipziger Str. 123, 36039 Fulda, Germany; 2 Division for Allergy, Pneumology and Cystic Fibrosis, Department for Children and Adolescence, Goethe University, Theodor-Stern-Kai 7, Frankfurt/Main, Germany; 3 Faculty of Biological Sciences, Goethe University, Max-von-Laue-Straße 13, Frankfurt/Main, Germany

**Keywords:** Omega-3, Omega-6, LCPUFA, Asthma, Fatty acid profiles, Gas-chromatography

## Abstract

**Background:**

The immune-modulating potential of long-chain polyunsaturated fatty acids (LCPUFAs) based on their conversion into lipid mediators in inflammatory situations has been proven by several studies. Respecting the immune-modulative role of lipid mediators in bronchoconstriction, airway inflammation and resolution of inflammatory processes, LCPUFAs play an important role in asthma. To design a disease-specific and most beneficial LCPUFA supplementation strategy, it is essential to understand how asthma alters LCPUFA profiles. Therefore, this study characterizes the alterations of LCPUFA profiles induced by allergic asthma. In addition, this study explores whether a simple eicosapentaenoic acid (EPA) alone or a specific combined LCPUFA supplementation could restore imbalanced LCPUFA profiles.

**Methods:**

Mice were sensitized with a daily dose of 40 μg house dust mite (HDM)-extract in a recall model and fed with either normal diet, EPA or a specific combined (sc)-LCPUFA supplementation containing EPA, docosahexaenoic acid (DHA), γ -linolenic acid (GLA) and stearidonic acid (SDA) for 24 days. After recall with HDM, mice were sacrificed and blood and lung tissue were collected. Fatty acid profiles were determined in plasma, blood cells and lung cells of asthmatic mice by capillary gas-chromatography.

**Results:**

In lung cells of asthmatic mice, arachidonic acid (AA, *p* < 0.001) and DHA (*p* < 0.01) were increased while dihomo-γ-linolenic acid (DGLA, *p* < 0.05) was decreased. EPA supplementation increased only EPA (*p* < 0.001) and docosapentaenoic acid (DPA, *p* < 0.001), but neither DGLA nor DHA in lung cells of asthmatic mice. In contrast, a specific combined dietary supplementation containing n-3 and n-6 LCPUFAs could decrease AA (*p* < 0.001), increase EPA (*p* < 0.001), DPA (*p* < 0.001) and DHA (*p* < 0.01) and could reverse the lack of DGLA (*p* < 0.05).

**Conclusions:**

In summary, allergic asthma alters LCPUFA profiles in blood and lung tissue. In contrast to the EPA supplementation, the distinct combination of n-3 and n-6 LCPUFAs restored the LCPUFA profiles in lung tissue of asthmatic mice completely. Subsequently, sc-LCPUFA supplementation is likely to be highly supportive in limiting and resolving the inflammatory process in asthma.

**Electronic supplementary material:**

The online version of this article (10.1186/s12944-018-0947-6) contains supplementary material, which is available to authorized users.

## Background

Allergic asthma is characterized by chronic respiratory eosinophilic and polymorphonuclear inflammation, airway hyperresponsiveness (AHR) and airway remodeling affecting more than 300 million people worldwide [1, 2]. Lipid mediators derived from omega-3 (n-3) and omega-6 (n-6) long-chain polyunsaturated fatty acids (LCPUFAs) play a key role in the initiation, maintenance and resolution of these inflammatory reactions [3].

Upon allergen exposure such as house dust mite (HDM) [4], LCPUFAs are released out of the cell membrane phospholipids by phosphatidylcholin-2-acylhydrases (PLA_2_) to be converted into lipid mediators through oxygenation by cyclooxygenases and lipoxygenases. While pro-inflammatory lipid mediators derive from n-6 arachidonic acid (AA) specialized pro-resolving mediators (SPMs) which can restore immune-homeostasis and induce broncho-protective mechanisms in the lung [5] mainly derive from n-3 LCPUFAs such as eicosapentaenoic acid (EPA), docosapentaenoic acid (DPA) and docosahexaenoic acid (DHA). In asthma, AA-derived prostaglandins, leukotrienes and thromboxanes function as pro-inflammatory molecules and potent bronchoconstrictors [6]. EPA-, DPA- and DHA-derived SPMs promote resolution processes and they have been described to reduce eosinophilic accumulation in tissue, Th2 cytokine production and AHR due to an enhanced allergen phagocytosis and clearance by macrophages [3, 7–10].

A direct correlation of the inflammatory situation in the bronchoalveolar-system of the lung and the n-6/n-3 status of asthmatic patients was shown in several studies [11, 12] indicating the necessity of dietary LCPUFA supplementations. And indeed, there has been a lot of data showing the positive effects of n-3 LCPUFA supplementations, such as EPA and DHA [13–15]. However, there has been also controversial data on the beneficial effects of n-3 LCPUFAs in asthma [16] which might be due to an incomplete analysis of LCPUFA profiles in blood and lung tissue leading to an insufficient restoration of LCPUFA profiles by distinct supplementation strategies.

The aim of this study was to characterize whether and how allergic asthma alters LCPUFA profiles in plasma, blood and lung cells of an in vivo asthma mouse model [17]. Thereby, the study should indicate immune response-initiated lipid metabolism activation and reflect the specific requirement of dietary supplementation of LCPUFAs in order to balance LCPUFA profiles. Considering imbalanced lipid metabolic pathways, we investigated further whether a specific combined (sc)-LCPUFA-supplementation, containing EPA, DHA, γ –linolenic- (GLA) and stearidonic acid (SDA) could modify and reverse more beneficially LCPUFA profile alterations in asthma than a usual n-3 LCPUFA supplementation, such as EPA. Thus, we supplemented mice with either EPA alone or a sc-LCPUFA blend to prove which supplementation strategy is capable to influence functional LCPUFA profiles of asthmatic mice more beneficially with the aim to support the lipid mediator dependent resolution processes of chronic inflammation.

## Material & Methods

### Mice keeping

All animal procedures were performed according to protocols approved by the German Animal Subjects Committee (Gen.Nr.FK/1036). Female C57BL/6 mice (6 to 8 weeks of age, purchased from Charles River Laboratories, Wilmington, USA) were housed in groups of five mice in stainless steel cages under sterile conditions. Mice were maintained in an air-conditioned room at the university hospital in Frankfurt/Main (Zentrale Forschungseinrichtung) with an alternating 12 h light/dark cycle.

### Allergic asthma mouse model

**Fig. 1:**
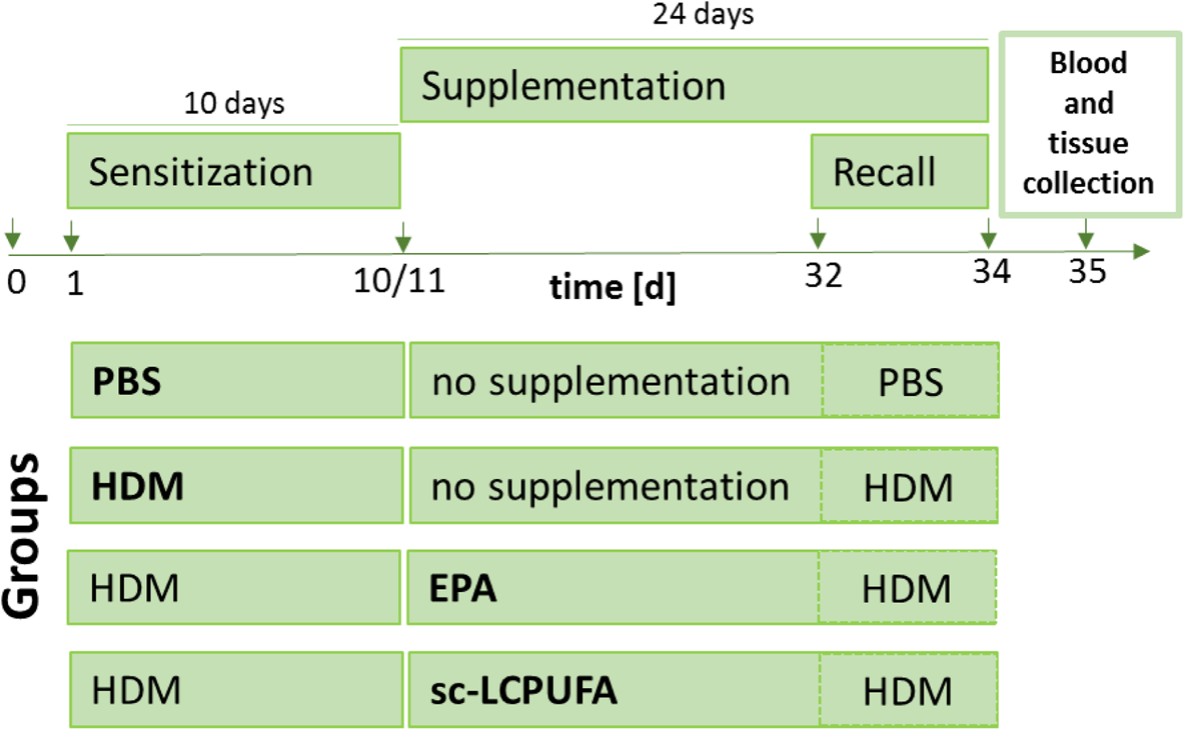
Experimental design. C57BL/6 mice were sensitized with HDM (or PBS as control) for ten consecutive days (day (d)1-d10). After sensitization, mice received either no supplementation (PBS control and HDM group) or EPA or sc-LCPUFA supplementation (EPA and sc-LCPUFA group) for 24 days from d11 to d34. Furthermore, all mice received repetitive doses of HDM (or PBS) from d32 to d34 representing a realistic recall model. On d35, blood and lung tissue was collected and fatty acid profiles were determined by gas-chromatography. All four treatment groups (PBS, HDM, EPA and sc-LCPUFA) were statistically analyzed by using One-Way ANOVA with Dunnet’s post-hoc analysis against the disease group (HDM)

As illustrated in Fig. 1, each mice was sensitized by house dust mite (HDM; *Dermatophagoides pteronyssinus)* extract (Greer, New York City, USA) diluted to a final concentration of 1.6 mg/mL in PBS (phosphate-buffered saline, pH = 7.4, Life Technologies, Darmstadt, Germany) for 10 consecutive days. At the start, the mice were anesthetized with isoflurane (initial dose: 5%, maintenance dose: 2.5%, Baxter, Unterschleißheim, Germany) to suppress the swallowing reflex. Thereafter, they either received intranasally 25 μL HDM (equal to 40 μg) or 25 μL PBS as control. After a resting period of 21 days, the mice received repetitive doses of HDM and PBS respectively for three days (Recall model, according to Cates et al. [17]). On day 35, 24 h after the last HDM administration, mice were sacrificed under anesthesia (Xylazin (10 mg/kg BW; Bayer Vital, Leverkusen, Germany) and Ketanest (100 mg/kg BW; CuraMed GmbH, Karlsruhe, Germany) by cardiac withdrawal. Blood was collected in EDTA-microvettes (Sarstedt, Nümbrecht, Germany) and immediately after the withdrawal, the samples were centrifuged, separated into plasma and blood cells and stored at − 80 °C until further preparation. Lungs were flushed with PBS through the pulmonary artery to wash out the erythrocytes. After this, lungs were removed and stored at − 80 °C until further measurement.

### Dietary supplementation

All mice were fed with water and standard lab chow with a fat content of 6% fat (C 14:0: 0.01%; C 16:0: 0.68%; C 16:1: 0.04%; C 18:0: 0.22%; C 18:1: 1.44%; C 18:2: 3.21%; C 18:3: 0.37%; C 20:0: 0.03%; C 20:1: 0.01%) (Ssniff, Soest, Germany) *ad libitum*. Moreover, the experimental diet (EPA or sc-LCPUFA) was applied orally by a feeding needle (canula, 0.70 × 30 mm, LL, curved, Knopf C, Robert Helwig GmbH, Berlin, Germany) connected to a 1 mL syringe (Becton Dickinson, Frankfurt/Main, Germany) for 24 days. The mice were started to be fed on day 11 after 10 days of HDM sensitization (see Fig. 1). EPA supplementation contained 1000 mg/kg BW per day EPA (*PlusEPA*, Minami Nutrition, Aartselaar, Belgium). The specific combined (sc)-LCPUFA supplementation was obtained by blending three different dietary oils by a ratio of 1:1:2 (v:v:v) *(PlusEPA*, Minami Nutrition, Aartselaar, Belgium; *EPA/DHA/GLA,* Peak Performance Products S.A., Grevenmacher, Luxemburg; *Echiomega*, Igennus Healthcare Nutrition, Cambridge, UK). Daily doses of the blend contained 1000 mg/kg BW EPA, 229.6 mg/kg BW DHA, 246.0 mg/kg BW GLA and 200.9 mg/kg BW SDA.

During supplementation, EPA or the sc-LCPUFA blend was freshly mixed on a daily basis and diluted in 0.5% (w:v) gum arabic solution (gum arabic powder, Carl Roth, Karlsruhe, Germany; sterile water) to a final volume of 200 μL per dose to assure that a defined and precise application volume was given. Just before administration, emulsions were homogenized using an ultrasonic homogenizer (Sonopuls, Bandelin, Berlin, Germany).

### Capillary gas-chromatographic analysis of the fatty acid profiles

Total lipids of lung tissue as well as separated plasma and blood cells were extracted according to Bligh and Dyer [18]. In preparation, lung tissue was homogenized and erythrocytes were lysed with ACK (Ammonium-Chloride-Potassium) lysing buffer (Sigma-Aldrich, Taufkirchen, Germany) to assure sole and proper extraction. For fatty acid methyl ester (FAME) derivatization, the total lipid fraction was completely dried using an evaporator (Liebisch Labortechnik GmbH, Bielefeld, Germany) with nitrogen (AlphaGaz 1, Air Liquide Deutschland GmbH, Düsseldorf, Germany) and then derivatized in accordance with the method of Kohn et al. [19]. After derivatization, the organic solvent was completely dried and FAMEs were resolved in hexane (Sigma-Aldrich, Taufkirchen, Germany) for measurement. The fatty acid profiles were analyzed by capillary gas-chromatography (Trace 1300, Thermo Scientific, Dreieich, Germany) which was equipped with an autosampler AS1310 (Thermo Fisher Scientific, Dreieich, Germany). The chromatographic separation of fatty acids was achieved using a capillary pre-column GuardGOLD, length: 2 m, i.d.: 0.25 mm (Thermo Fisher Scientific, Dreieich, Germany) and a capillary column named TRACE TR-FAME (70% cyanopropyl polysilphenylene siloxane, length: 60 m, i.d.: 0.25 mm, film thickness: 0.25 μm, Thermo Fisher Scientific, Dreieich, Germany).

The gas-chromatographic conditions were the following: injector (SSL): 250 °C, splitless and carrier gas: helium (purification 99%. Air Liquide Deutschland GmbH, Düsseldorf, Germany) at a flow of 20 mL/min. Compounds were detected by a flame-ionization detector (FID) at 250 °C. Fatty acids were identified by their retention times (RT) compared with the RT of fatty acids in the external standard. External standard was composed of Supelco 37-Component FAME Mix (Sigma-Aldrich, Taufkirchen, Germany) plus seven methylated single standards: vaccenic acid (VAC; C18:1n-7), stearidonic acid (SDA; C18:4n-3), nonadecanoic acid (C19:0; as internal standard), mead acid (MA; C20:3n-9), adrenic acid (ADA; C22:4n-6), docosapentaenoic acid (DPA; C22:5n-3) and palmitoleic acid (C16:1n-7 t). Standards of VAC, C19:0, MA, ADA and DPA were all purchased from Sigma-Aldrich (Taufkirchen, Germany), whereas SDA and C16:1n-7 t were purchased from Biomol (Hamburg, Germany). The total fatty acid standards qualified 95.00 ± 0.20% of all fatty acids detected.

### Statistics

Data are displayed as mean ± standard error of mean (SEM) with *n* = 5–10. The gas-chromatographic total peak areas were exported from Chromeleon7™ (Thermo Fisher, Waltham, USA) and were evaluated by Excel and GraphPad Prism 5 and 7 (GraphPad Software, La Jolla, USA). Comparisons between the four groups (PBS, HDM, EPA and sc-LCPUFA) were performed by using One-Way ANOVA with Dunnet’s post-hoc analysis against the disease group (HDM). Differences were considered as statistically significant when *P*-values < 0.05. The following symbols indicate significant *P*-values: ^∗^ *P* < 0.05, ^∗∗^*P* < 0.01 and ^∗∗∗^*P* < 0.001.

## Results

**Fig. 2:**
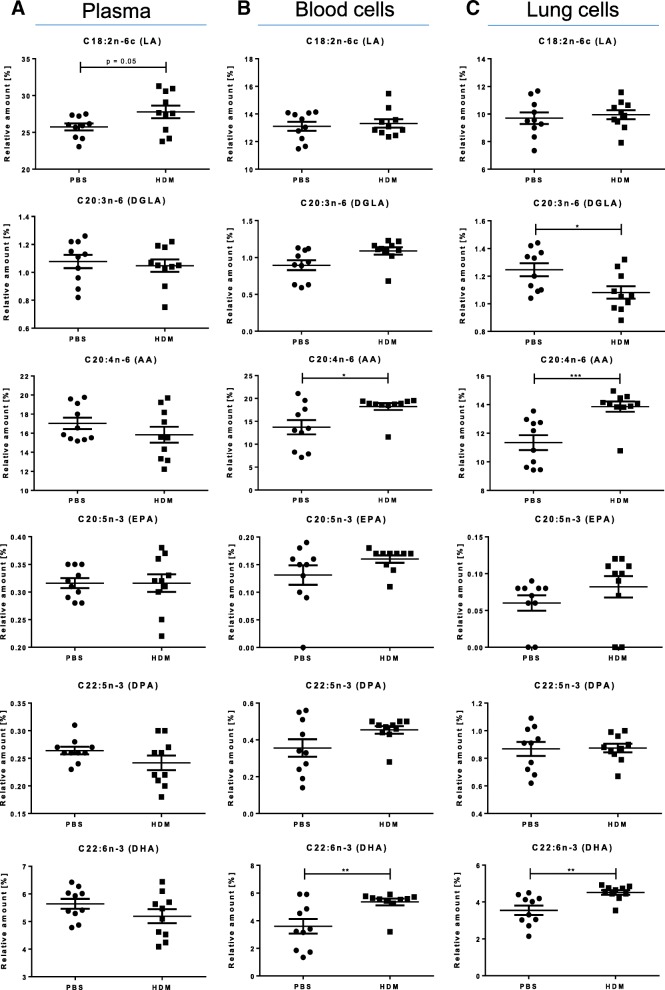
Allergic asthma altered LCPUFA profiles (Comparison: PBS vs. HDM). Relative amounts of n-3 and n-6 fatty acids LA, DGLA, AA, EPA, DPA and DHA in (**a**) murine plasma (**b**) blood cells and (**c**) lung cells on day 35 of control mice which received PBS (PBS) and asthmatic mice which received HDM (HDM) during sensitization and recall. Data is represented by mean ± SEM out of *n* = 10 for each group. Statistical tests were performed by using One-Way ANOVA with Dunnet’s post-hoc analysis against the disease group (HDM) to statistically compare the results of all four treatment groups (PBS, HDM, EPA and sc-LCPUFA) shown in this article. *P*-Values were considered as statistically significant *P < 0.05, **P < 0.01, ***P < 0.001

**Fig. 3:**
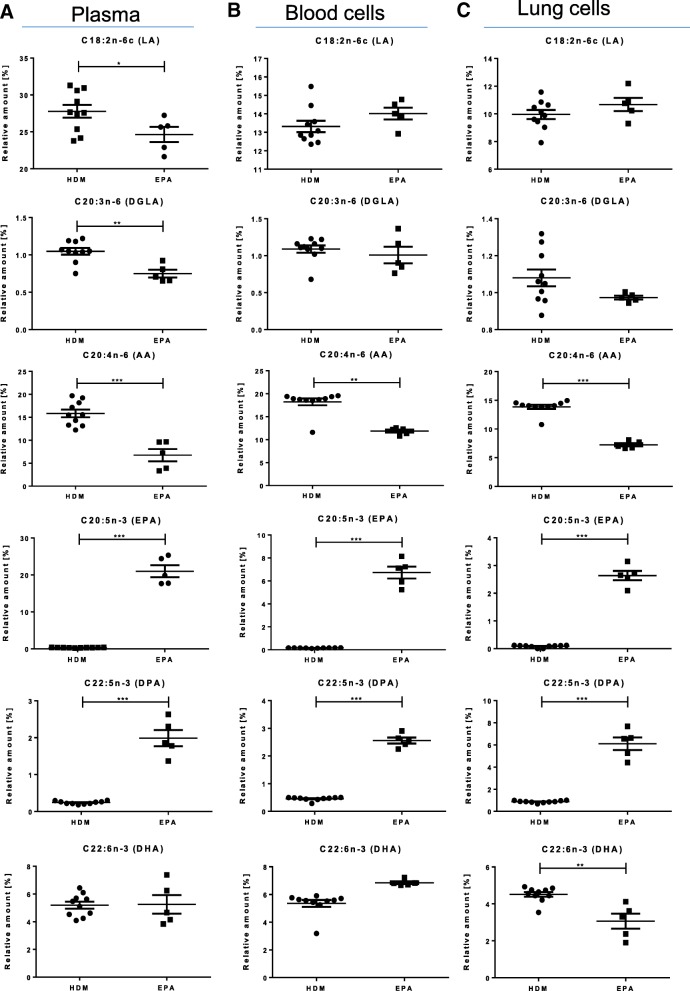
EPA supplementation could not completely reverse disease-specific LCPUFA alterations (Comparison: HDM vs. EPA). Relative amounts of n- 3 and n-6 fatty acids LA, DGLA, AA, EPA, DPA and DHA in (**a**) murine plasma (**b**) blood cells and (**c**) lung cells regarding asthmatic (HDM) and EPA supplemented asthmatic (EPA) mice on day 35. Data is represented by mean ± SEM out of *n* = 5–10 for each group. Statistical tests were performed by using One-Way ANOVA with Dunnet’s post-hoc analysis against the disease group (HDM) to statistically compare the results of all four treatment groups (PBS, HDM, EPA and sc-LCPUFA) shown in this article. P-Values were considered as statistically significant *P < 0.05, **P < 0.01, ***P < 0.001

**Fig. 4:**
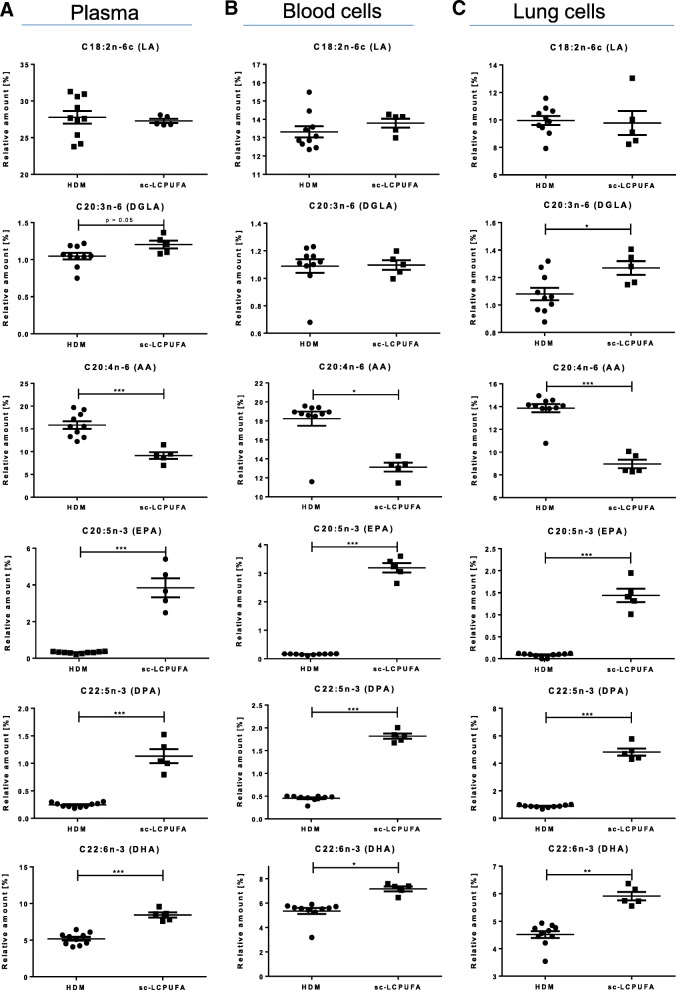
Sc-LCPUFA supplementation could restore asthma-specific altered LCPUFA profiles (Comparison: HDM vs. sc-LCPUFA). Relative amounts of n-3 and n-6 fatty acids LA, DGLA, AA, EPA, DPA and DHA in (**a)** murine plasma (**b)** blood cells and (**c)** lung cells of asthmatic (HDM) and sc-LCPUFA supplemented (sc-LCPUFA) asthmatic mice on day 35. Data is represented by mean ± SEM out of n = 5–10 for each group. Statistical tests were performed by using One-Way ANOVA with Dunnet’s post-hoc analysis against the disease group (HDM) to statistically compare the results of all four treatment groups (PBS, HDM, EPA and sc-LCPUFA) shown in this article. *P*-Values were considered as statistically significant *P < 0.05, **P < 0.01, ***P < 0.001

To investigate the alterations of LCPUFA profiles in allergic asthma and the incorporation of LCPUFAs, the relative amount of distinct fatty acid species was determined in murine plasma as well as in blood and lung cells of mice by capillary gas-chromatography on day 35 (see Fig. 1). Therefore, mice were first sensitized to HDM (or PBS as control) for ten consecutive days (day (d)1-d10) followed by either no supplementation (PBS and HDM group) or EPA or sc-LCPUFA supplementation (EPA and sc-LCPUFA group) for 24 days from d11 to d34. Thereafter, all mice received repetitive doses of HDM (or PBS as control) from d32 to d34 representing a realistic recall model. To show disease- and supplementation specific differences (Figs. 2, 3, 4), results were statistically analyzed by using One-Way ANOVA with Dunnet’s post-hoc analysis against the disease group (HDM) and always depicted compared to HDM. Complete data of all four treatment groups (PBS, HDM, EPA and sc-LCPUFA) can be found in the Additional files 1, 2, 3.

### Allergic asthma influences LCPUFA profiles of murine blood and lung cells

In Fig. 2, LCPUFA profiles of plasma, blood and lung cells of control (PBS) and asthmatic mice (HDM) are shown. For plasma, (Fig. 2a) there were no significant changes found although there was a trend that LA was increased in the HDM-sensitized mice (controls: 25.74 ± 0.45%; asthmatic: 27.78 ± 0.82%, *p* = 0.05). In contrast to plasma, the relative amount of AA and DHA levels were significantly increased in blood and lung cells (Fig. 2b and c) of asthmatic mice (blood cells: AA: from 13.71 ± 1.48 to 18.24 ± 0.71%, *p* < 0.05; DHA: from 3.59 ± 0.50 to 5.35 ± 0.23%, *p* < 0.01; lung cells: AA: from 11.34 ± 0.49 to 13.86 ± 0.34%, *p* < 0.001; DHA: from 3.55 ± 0.24 to 4.51 ± 0.12%, *p* < 0.01). Interestingly, DGLA, a precursor for an anti-inflammatory lipid mediator named PGE_1_, was significantly diminished in lung cells of asthmatic mice (DGLA: from 1.25 ± 0.05 to 1.08 ± 0.04%, *p* < 0.05).

To ensure that the alterations of LCPUFA profiles in asthma can be clearly attributed to the induced asthma, the allergic inflammatory situation was determined in bronchoalveolar lavage fluid (BALF) which revealed an elevated migration of eosinophils from 0.00 ± 0.00% to 57.87 ± 3.08%. Additionally, there was an enhanced Th2-immune response driven release of cytokine IL-5 from 135.5 ± 37.0 fg/mL to 10,571.6 ± 3698.8 fg/mL (*p* < 0.05) (data not shown; will be published elsewhere in detail).

### EPA supplementation could not completely reverse asthma-specific alterations of LCPUFA profiles in asthmatic mice

To investigate how a common n-3 LCPUFA supplementation would affect LCPUFA profiles in asthma, LCPUFA profiles of plasma, blood and lung cells were determined in asthmatic mice supplemented with EPA for 24 days after sensitization (see Fig. 1). With regards to functional LCPUFAs (Fig. 3a), EPA significantly increased the relative amount of EPA as well as DPA and significantly decreased LA, DGLA and AA in plasma of asthmatic mice (EPA: from 0.32 ± 0.02% to 21.01 ± 1.63%, *p* < 0.001; DPA: from 0.24 ± 0.01% to 1.99 ± 0.22%, *p* < 0.001; LA: from 27.78 ± 0.82% to 24.65 ± 1.03%, *p* < 0.05; DGLA: from 1.05 ± 0.04% to 0.75 ± 0.05%, *p* < 0.01; AA: from 15.84 ± 0.79% to 6.75 ± 1.35%, *p* < 0.001).

In blood and lung cells (Fig. 3b and c), EPA supplementation reduced AA and increased EPA and DPA, (blood cells: AA: from 18.24 ± 0.71% to 11.86 ± 0.33%, *p* < 0.01; EPA: from 0.16 ± 0.01% to 6.73 ± 0.51%, *p* < 0.001; DPA: from 0.45 ± 0.02% to 2.56 ± 0.11%, *p* < 0.001; lung cells: AA: from 13.86 ± 0.34% to 7.24 ± 0.29%, *p* < 0.001; EPA: from 0.08 ± 0.01% to 2.64 ± 0.17%, *p* < 0.001; DPA: from 0.87 ± 0.03% to 6.10 ± 0.57%, *p* < 0.001). However, EPA supplementation diminished the amount of DHA significantly from 4.51 ± 0.12% to 3.06 ± 0.41% (*p* < 0.01). Furthermore, EPA did not increase the level of functional n-6 LCPUFA DGLA but even diminished the relative amount of it in plasma from 1.05 ± 0.04% to 0.75 ± 0.05% (*p* < 0.01).

### Disease-specific alterations could be restored by a specific combined (sc)-LCPUFA supplementation

The sc-LCPUFA supplementation increased the functional n-3 LCPUFAs EPA, DPA and DHA in plasma, blood as well as in lung cells of asthmatic mice (Fig. 4a–c: plasma: EPA: from 0.32 ± 0.02% to 3.85 ± 0.52%, *p* < 0.001; DPA: from 0.24 ± 0.01% to 1.13 ± 0.13%, *p* < 0.001; DHA: from 5.19 ± 0.24% to 8.43 ± 0.35%, *p* < 0.001; blood cells: EPA: from 0.16 ± 0.01% to 3.19 ± 0.16%, *p* < 0.001; DPA: from 0.45 ± 0.02% to 1.82 ± 0.06%, *p* < 0.001; DHA: from 5.35 ± 0.23% to 7.16 ± 0.20%, *p* < 0.05; lung cells: EPA: from 0.08 ± 0.01% to 1.44 ± 0.15%, *p* < 0.001; DPA: from 0.87 ± 0.03% to 4.82 ± 0.26%, *p* < 0.001; DHA: from 4.51 ± 0.12% to 5.91 ± 0.15%, *p* < 0.01). Furthermore, the increase of AA in mice with allergic asthma could be reduced and AA levels were significantly lower in plasma as well as in blood and lung cells (AA: plasma: from 15.84 ± 0.79% to 9.16 ± 0.73%, *p* < 0.001; blood cells: from 18.24 ± 0.71% to 13.13 ± 0.47%, *p* < 0.05; lung cells: from 13.86 ± 0.34% to 8.96 ± 0.38%, *p* < 0.001). Moreover, although DGLA was reduced significantly in lung cells of asthmatic mice, it was reobtained to the level of healthy mice by a significant increase (from 1.08 ± 0.04% to 1.27 ± 0.05%, *p* < 0.05).

## Discussion

The beneficial effects of LCPUFAs on chronic airway inflammation are discussed controversially in the literature [13–16]. Therefore, our aim was to identify disease-dependent LCPUFA profile alterations in asthma and to investigate the effects of specific combined LCPUFAs (sc-LCPUFA) compared to solely EPA supplementation on the fatty acid profile, in blood and lung tissue of asthmatic mice (see Fig. 1).

In contrast to plasma, there was a significant increase of AA and DHA in blood and lung cells and a significant decrease of DGLA in lung cells of asthmatic mice (Fig. 2b–c) which could indicate a constant release of LCPUFAs from cell membranes and a consequent replenishment from plasma. These findings are consistent with current literature. Previous studies have revealed an increased PLA_2_ activity due to the stimulation of Toll-like-receptor-4 and other receptors that are triggered upon allergen exposure, such as HDM, in allergic asthma [4]. Thus, sensitization of mice with HDM results in destruction of homeostasis and leads in an unbalanced LCPUFA content within cells. Likewise, Calabrese et al. described higher AA contents in eosinophils of human asthmatics [20]. In summary, fatty acid profiles of asthmatic mice reveal that LCPUFAs accumulate significantly according to their availability in plasma which results in a higher replenishment of AA and DHA without any LCPUFA supplementations.

Using a common n-3 EPA supplementation, our results showed that EPA increased EPA and DPA and reduced AA in plasma, blood and lung cells, but did not elevate DHA levels in lung cells (Fig. 3a–c). Whereas EPA can be rapidly converted into DPA by elongation [21], the conversion of DPA to DHA is limited due to the Δ6-desaturase activity leading only to a tendentious increase of DHA amounts in blood cells and even significantly decreased levels of DHA in lung cells. However, DHA and its pro-resolving mediators are well described potential therapeutic agents in asthma by diminishing eosinophils and pro-inflammatory mediators and by promoting macrophage clearance of allergen [7]. Hence, it seems to be critical to not only increase EPA and DPA, but also DHA to maximize the beneficial effects of LCPUFA supplementations in asthma.

Regarding the lack of the functional n-6 LCPUFA DGLA in lung cells of asthmatic mice, EPA did not reobtain DGLA, but decreased its relative amount even further (Fig. 3). Therefore, EPA supplementation was not able to compensate the lack of DGLA in asthma, although EPA is described to inhibit the metabolization of DGLA to AA [22]. Since EPA supplementation significantly decreased DHA in lung cells and did not increase DGLA levels compared to asthmatic mice shown in Fig. 3a–c, these results could point out that EPA supplementation alone might even exacerbate the unbalanced LCPUFA situation in asthmatic mice. However, DGLA is a precursor for an anti-inflammatory eicosanoid, named prostaglandin (PG) E_1_ [23], and it inhibits the formation of AA to leukotriene (LT) B_4_ effectively [24]. Regarding the noteworthy role of DGLA and its significant decrease in chronically inflamed lung cells of asthmatic mice, it might be critical to not only increase the n-3 LCPUFA pool but also to reobtain the DGLA amount. Hence, we suggest to elevate DGLA by LCPUFA supplementation.

We addressed the need of DGLA by supplementing the naturally occurring GLA, which can be rapidly converted into DGLA by elongation [25]. To inhibit the conversion of DGLA into AA further and to support the accumulation of DGLA, we also added stearidonic acid (SDA) to the sc-LCPUFA supplementation blend [22, 26]. Hence, we supplemented asthmatic mice with the sc-LCPUFA blend containing not only EPA but also DHA, GLA and SDA. Thereby, we could show that the sc- LCPUFA blend influenced both n-3 and n-6 fatty acid metabolism beneficially. EPA, DPA and DHA were significantly increased and AA was significantly decreased in plasma, blood and lung cells by the sc-LCPUFA supplementation (Fig. 4a–c). Moreover, the sc-LCPUFA blend reobtained DGLA levels in lung cells of asthmatic mice (Fig. 4c).

In summary and according to our findings, LCPUFA supplementations in asthma should increase EPA, DPA and DHA to maximize the pool of SPMs, reobtain DGLA levels and decrease AA to balance disease-specific LCPUFA alterations and to influence the clinical outcome of asthma most beneficially. Thereby, it would be conceivable to possess the most possible immune modulatory effects respecting influences of both n-3 and n-6 LCPUFAs and their interactions as we described in our review [27]. Based on our findings about disease-specific LCPUFA profile alterations, we suggest the use of individual and disease-dependent specific combined LCPUFA supplementations to reverse disease-specific LCPUFA alterations and to promote LCPUFA homeostasis. This is likely to be more supportive in order to support the lipid mediator dependent resolution process compared to single LCPUFA supplementations.

## Conclusions

In summary, AA and DHA were increased significantly in blood and lung cells of asthmatic mice suggesting replenishment of LCPUFAs from plasma into the cell membranes following a continuing release of LCPUFAs upon PLA_2_ activity in chronic airway inflammation. Whereas AA and DHA were increased in asthma, DGLA, which is a precursor for predominantly anti-inflammatory mediators, was significantly decreased in lung cells of chronic asthmatic mice. Considering this, we showed that a specific combined dietary supplementation containing a combination of dietary n-3 and n-6 LCPUFAs EPA, DHA, GLA and SDA could increase EPA, DPA and DHA to maximize the pool of SPMs, reobtained DGLA levels and decreased AA in order to balance disease-specific LCPUFA alterations. In contrast, a common n-3 supplementation with EPA alone neither increased DHA nor DGLA in lung cells. Therefore, the asthma-specific LCPUFA combination should be the preferable option compared to single EPA supplementation. By recovering LCPUFA homeostasis in cellular membranes and ensuring biosynthesis of SPMs, the asthma specific LCPUFA combination is likely to be more supportive in limiting and resolving inflammatory processes in asthma.

## Additional files

Additional file 1: Fatty acid profiles of murine plasma. Fatty acid profiles of murine plasma of control (PBS) and asthmatic mice either non-supplemented (HDM) or supplemented with EPA (EPA) or sc-LCPUFA (sc-LCPUFA). Data are represented by mean ± SEM out of *n* = 5–10 for each group. (XLS 37 kb)

Additional file 2: Fatty acid profiles of murine blood cells. Fatty acid profiles of murine blood cells of control (PBS) and asthmatic mice either non-supplemented (HDM) or supplemented with EPA (EPA) or sc-LCPUFA (sc-LCPUFA). Data are represented by mean ± SEM out of n = 5–10 for each group. (XLS 37 kb)

Additional file 3: Fatty acid profiles of murine lung cells. Fatty acid profiles of murine lung cells of control (PBS) and asthmatic mice either non-supplemented (HDM) or supplemented with EPA (EPA) or sc-LCPUFA (sc-LCPUFA). Data are represented by mean ± SEM out of n = 5–10 for each group. (XLS 37 kb)

## Abbreviations

AA: Arachidonic acid
AHR: Airway hyperresponsiveness
BALF: Bronchoalveolar lavage fluid
DGLA: Dihomo-γ-linolenic acid
DHA: Docosahexaenoic acid
DPA: Docosapentaenoic acid
EPA: Eicosapentaenoic acid
FAME: Fatty acid methyl esters
GLA: γ-linolenic acid
HDM: House dust mite
LCPUFA: Long-chain polyunsaturated fatty acids
LT: Leukotrienes
n-3: Omega-3
n-6: Omega-6
PBS: Phosphate-buffered saline
PG: Prostaglandins
PLA_2_: Phosphatidylcholin-2-acylhydrases
RT: Retention time
SDA: Stearidonic acid
SPM: Specialized pro-resolving mediator
